# Upregulation of CD11b and CD86 through LSD1 inhibition promotes myeloid differentiation and suppresses cell proliferation in human monocytic leukemia cells

**DOI:** 10.18632/oncotarget.18564

**Published:** 2017-06-19

**Authors:** Jianwu Fang, Haiyan Ying, Ting Mao, Yanjia Fang, Yuan Lu, He Wang, Irene Zang, Zhaofu Wang, Ying Lin, Mengxi Zhao, Xiao Luo, Zongyao Wang, Yan Zhang, Chao Zhang, Wei Xiao, Yan Wang, Wei Tan, Zhui Chen, Chris Lu, Peter Atadja, En Li, Kehao Zhao, Jianfeng Liu, Justin Gu

**Affiliations:** ^1^ Key Laboratory of Molecular Biophysics of Ministry of Education, College of Life Science and Technology, Huazhong University of Science and Technology, Wuhan 430074, China; ^2^ China Novartis Institutes for BioMedical Research, Pudong New Area, Shanghai 201203, China

**Keywords:** LSD1, CD11b, CD86, myeloid differentiation, monocytic leukemia

## Abstract

LSD1 (Lysine Specific Demethylase1)/KDM1A (Lysine Demethylase 1A), a flavin adenine dinucleotide (FAD)-dependent histone H3K4/K9 demethylase, sustains oncogenic potential of leukemia stem cells in primary human leukemia cells. However, the pro-differentiation and anti-proliferation effects of LSD1 inhibition in acute myeloid leukemia (AML) are not yet fully understood. Here, we report that small hairpin RNA (shRNA) mediated LSD1 inhibition causes a remarkable transcriptional activation of myeloid lineage marker genes (CD11b/ITGAM and CD86), reduction of cell proliferation and decrease of clonogenic ability of human AML cells. Cell surface expression of CD11b and CD86 is significantly and dynamically increased in human AML cells upon sustained LSD1 inhibition. Chromatin immunoprecipitation and quantitative PCR (ChIP-qPCR) analyses of histone marks revealed that there is a specific increase of H3K4me2 modification and an accompanied increase of H3K4me3 modification at the respective CD11b and CD86 promoter region, whereas the global H3K4me2 level remains constant. Consistently, inhibition of LSD1 *in vivo* significantly blocks tumor growth and induces a prominent increase of CD11b and CD86. Taken together, our results demonstrate the anti-tumor properties of LSD1 inhibition on human AML cell line and mouse xenograft model. Our findings provide mechanistic insights into the LSD1 functions in controlling both differentiation and proliferation in AML.

## INTRODUCTION

Leukemia is a highly complex disorder of blood and bone marrow, and causes more deaths than any other cancer among children and young adults under the age of 20 [1]. It is characterized by inhibition of differentiation and promotion of oncogenic proliferation, usually leading to an uncontrolled abnormal cell proliferation [2, 3]. Acute leukemia, including acute lymphoblastic leukemia (ALL) and acute myeloid leukemia (AML), is the most common cancer affecting children under the age of 15. The 5-year survival rates for AML patients are only about 25% [4]. Therefore, new approaches are needed to provide valid therapeutic regimes that offer safe and reliable treatment.

Mixed lineage leukemia (MLL) is a genetically distinct form of acute leukemia that constitutes over 70% of infant acute leukemia and approximately 10% of adult AML [5]. MLL disease is characterized by chromosomal translocations affecting the MLL gene at 11q23, resulting in a variety of MLL fusion proteins [6–8]. In the disease-linked translocations, the catalytic histone H3K4 methyltransferase SET domain of the MLL protein is lost and the remaining MLL protein is fused to a variety of partners, including members of the AFF and ENL family of proteins such as AF4, AF5, AF6, AF9, AF10, SEPT5 and ENL, all of which are associated with super elongation complexes (SECs) [5, 9–11]. MLL-rearranged leukemia represents a particularly aggressive form of leukemia. AML Patients with MLL-rearrangement generally have poor prognoses and often suffer from early relapse after treatment with current standard induction therapies, a 7-day continuous infusion of cytarabine at the dosage of 100 or 200 mg/m^2^per day on days 1 to 7 and daunorubicin at 60 mg/m^2^ per day on days 1 to 3 [12, 13]. Thus, there is a pressing need for new treatment modalities for AML patients suffering from MLL rearrangement.

Of 80 different direct and 120 reciprocal MLL fusions, MLL-AF9 is capable of interacting directly, or indirectly, with one histone demethylase, LSD1 (also known as KDM1, AOF2, or BHC110) [1, 14, 15]. As a result, MLL-AF9 translocation products retain targeted gene recognition elements within the N terminal of the MLL protein, but also gain the ability to recruit LSD1 to these locations [9, 16–19]. LSD1 was initially discovered to specifically remove mono- and dimethyl groups from methylated histone H3 at lysine 4 (H3K4) to suppress gene expression [20–22]. Later, it was also found to demethylase the repressive mono- and di-methylated lysine 9 on histone H3 (H3K9) in an androgen-receptor-dependent manner in prostate cancer cells [23–25]. In addition, LSD1 and the orphan nuclear receptor estrogen-related receptor α (ERRα) coregulate several target genes involved in cell migration, which is mediated through H3K9 demethylation at the transcription start site (TSS) [26].

LSD1 is known to interact with the corepressor complex CoREST, containing RE1-silencing transcription factor (REST) and the histone deacetylases (HDAC) 1 and 2, which augments the gene repression activity of LSD1 [27]. Recent studies have shown increased LSD1 expression in AML (http://www.proteinatlas.org) [28]. Overexpression of the shortest isoform of LSD1 in hematopoietic stem cells (HSC) increased self-renewal potential but retained multi-differentiation ability, which synergized with genetic abnormalities in later stages to develop full-blown acute myeloid leukemia [29]. High LSD1 expression blocks differentiation and sustains the leukemogenic potential of the MLL-AF9 leukemia stem cells to confer a poor prognosis in AML [27, 30]. Furthermore, pharmacological inhibition of LSD1 results in induction of differentiation in both murine and primary human leukemia cells [1, 28]. In addition, AML cells show a higher sensitivity to all-trans-retinoic acid (ATRA) when ATRA was combined with LSD1 inhibition [2, 31]. Preclinical studies have revealed that pharmacological LSD1 inhibition can promote the expression of cell surface markers, including CD11b and CD86, associated with a differentiated immuno-phenotype in 12 of 13 AML cell lines [4, 32]. CD11b is a differentiation marker for cells of the myeloid-monocytic lineage [33]. B7-2 (CD86) is one of type I transmembrane proteins that were originally identified as ligands for CD28/CTLA-4, which are associated with T cell activation of immune system [34, 35]. Collectively, these reports strongly suggest that targeted knockdown of LSD1 might also induce monocyte to myeloid differentiation and attenuate tumor growth in human MLL-AF9 translocated AML. However, the anti-tumor effect of genetic inhibition of LSD1 in MLL-AF9 AML has not been fully understood. Toward this end, here we investigated the effects of genetic inhibition of LSD1 on cell differentiation and proliferation in human MLL-AF9 acute myeloid leukemia cells THP-1 (MLL-AF9) and Molm13 (MLL-AF9). We found that genetic inhibition of LSD1 promotes myeloid differentiation and inhibits cell proliferation *in vitro* and *in vivo*. Serials of validation studies revealed that genetic inhibition of LSD1 results in increased expressions of two myeloid differentiation markers (CD11b and CD86), reduced clonogenicity and decreased proliferation ability in THP-1 and Molm13 cells, while it has no any obvious effect in Jurkat (MLL-WT) cells. Histone 3 lysine methylation profiling by ChIP-qPCR further demonstrated that genetic inhibition of LSD1 increases H3K4me2 and H3K4me3 level on the promoters of CD11b and CD86. The same observation appears on the promoter of CD11b in Molm13 with pharmacological inhibition of LSD1. Additionally, we did not find a clear variation of H3K9me2 modification on those promoters in the same context, which indicates this increased expression of CD11b and CD86, is mainly due to H3K4 modification. Together, our findings underline the possible therapeutic potential of genetic inhibition of LSD1 in MLL-AF9 AML.

## RESULTS

### Knockdown of LSD1 reduces proliferation and clonogenicity of AML

**Figure 1:**
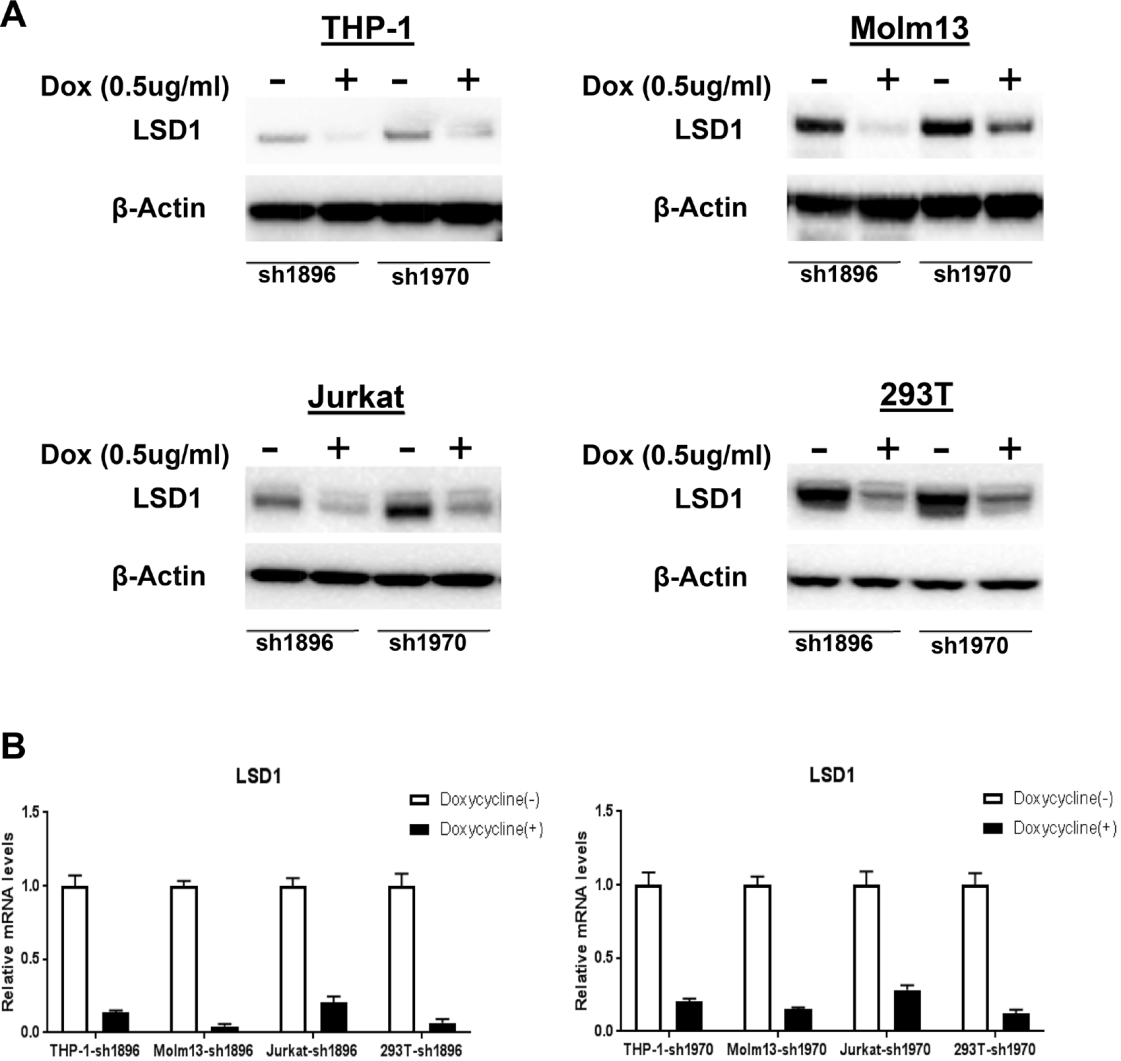

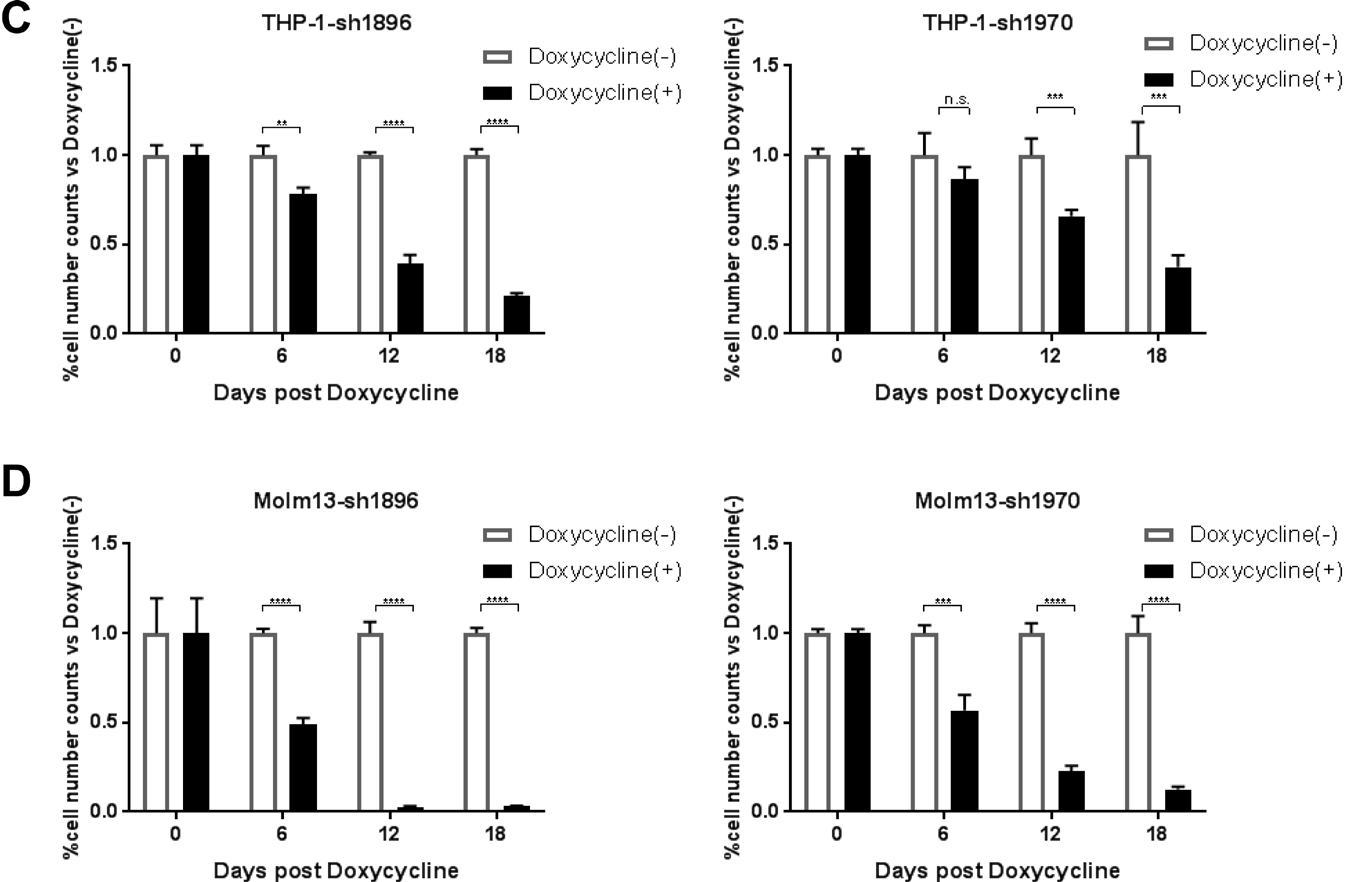

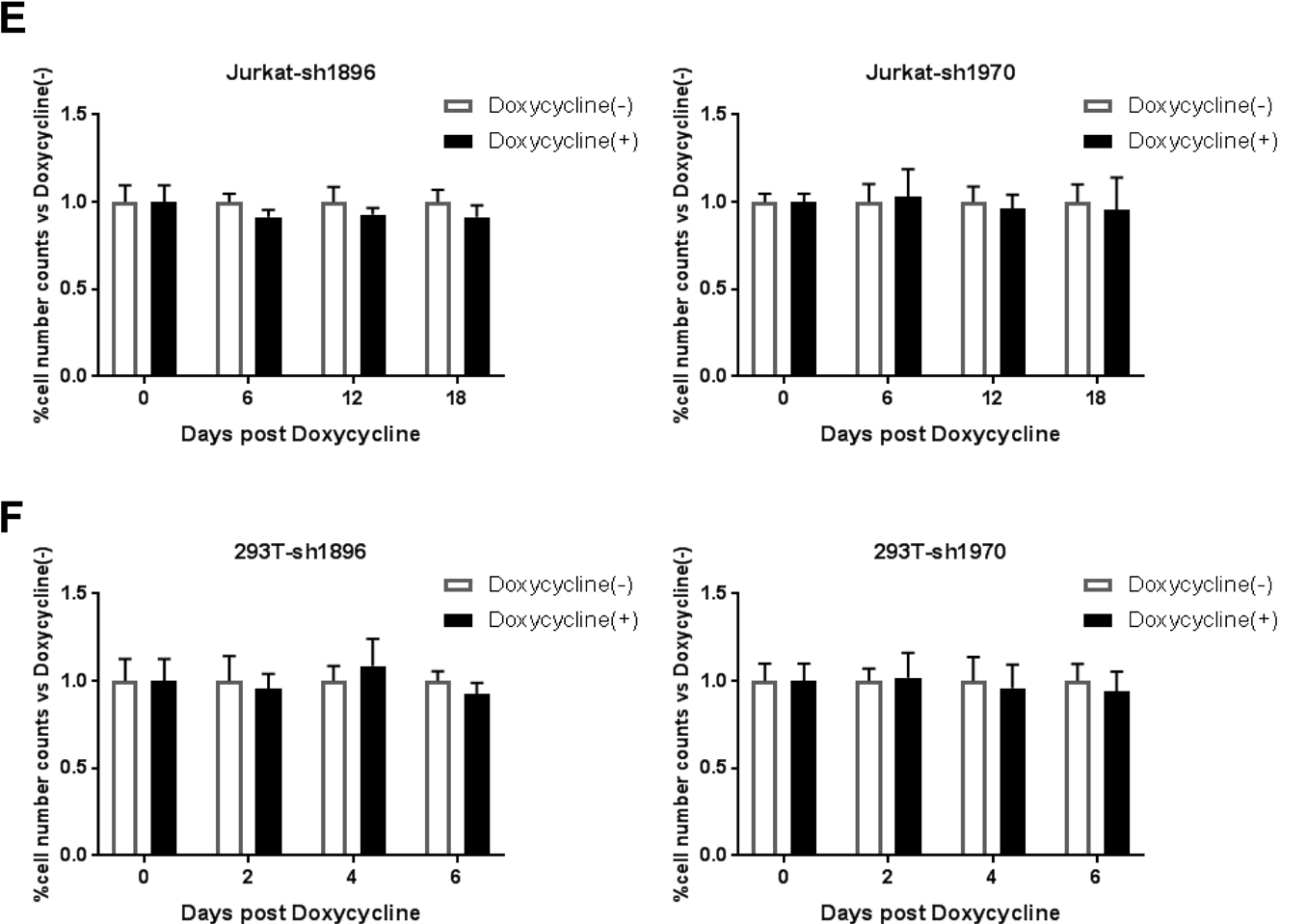

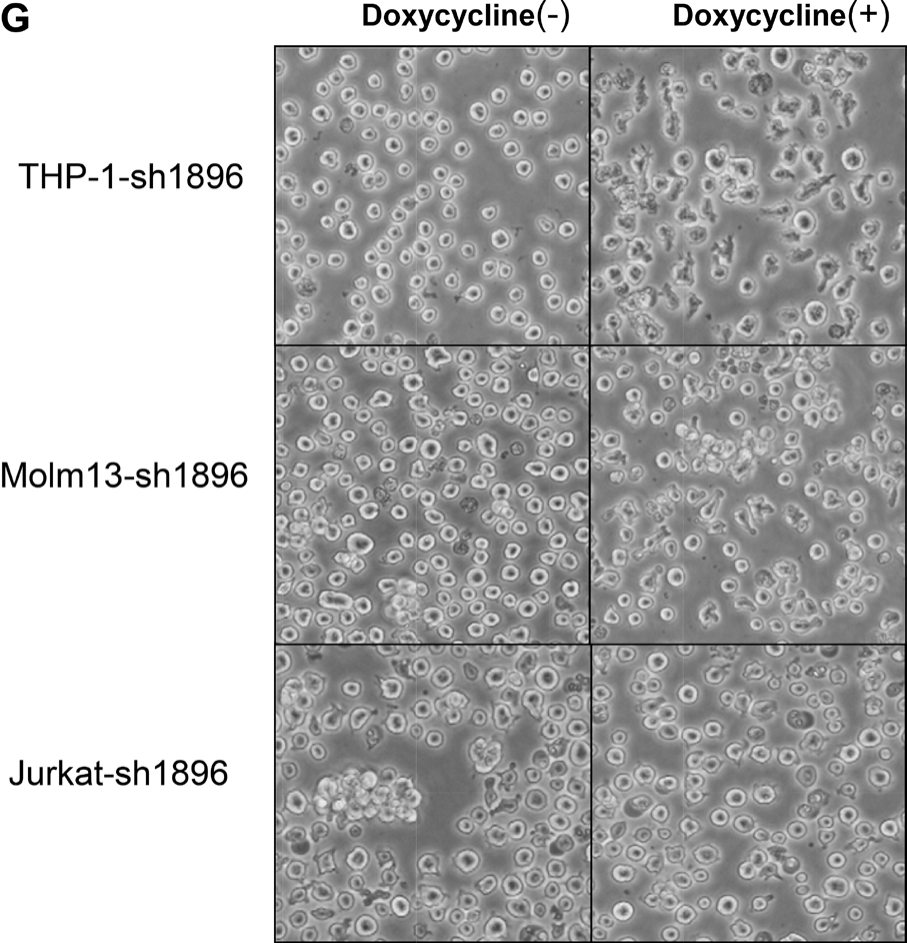

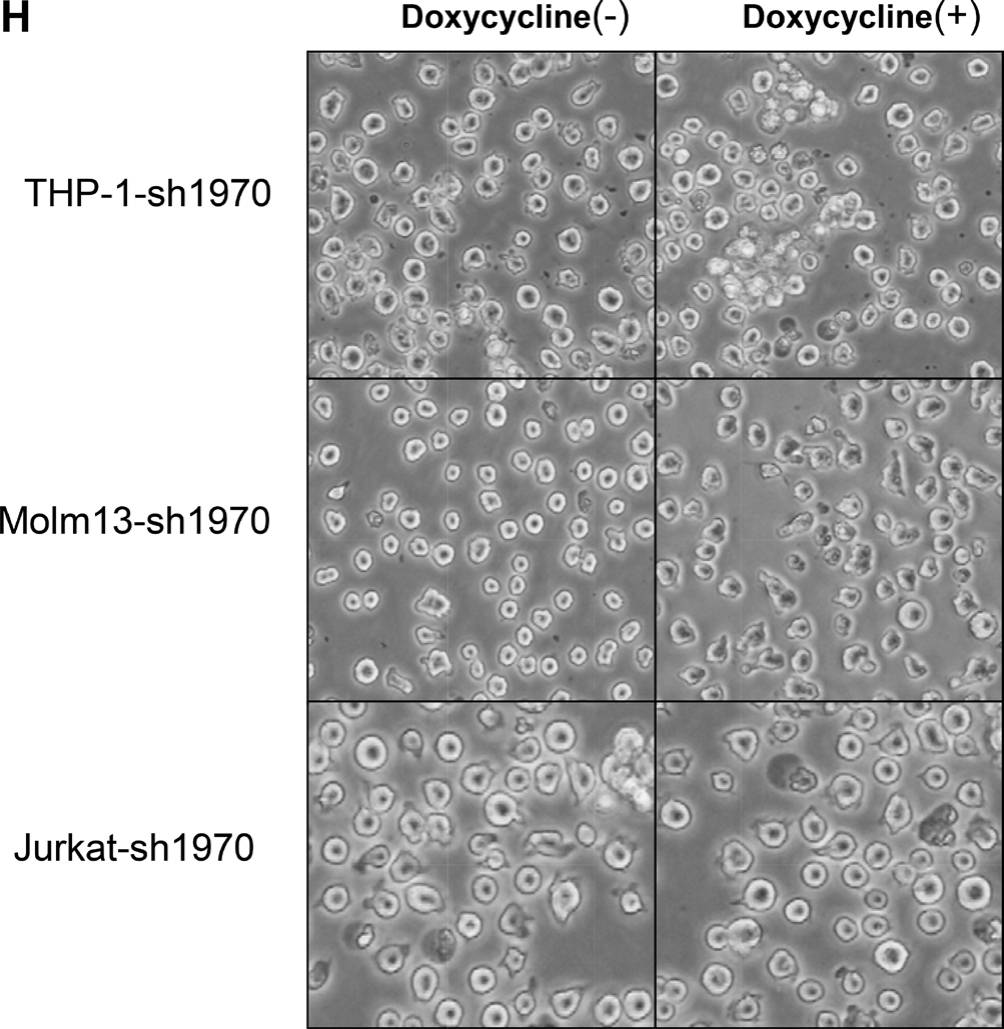

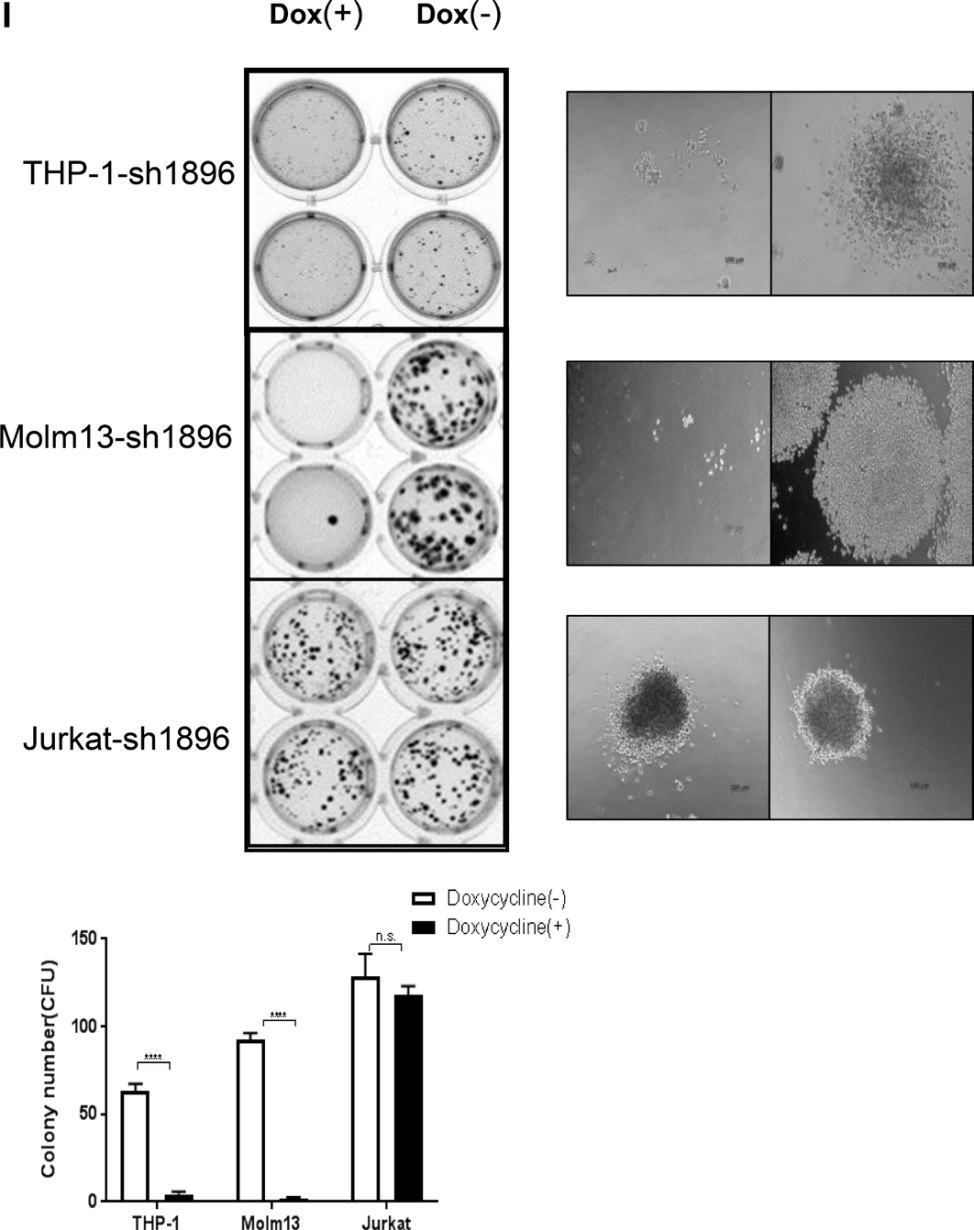
Knockdown of LSD1 blocks cell proliferation and clonogenicity *in vitro* (**A**) Western blotting analysis of 72 hours Dox (0.5 ug/ml) induced LSD1 Knockdown effect in pLenti6.3 V5-shRNA1896/1970 infected THP-1,Molm13,Jurkat and HEK293T (**B**) RT-PCR analysis of LSD1-shRNA Knockdown in pLenti6.3 V5-shRNA1896/1970 infected THP-1, Molm13, Jurkat and HEK293T. (**C, D, E, F**) Effects of Dox (0.5 ug/ml) induced LSD1 Knockdown effect on cell growth in pLenti6.3 V5-shRNA1896/1970 infected THP-1, Molm13, Jurkat and HEK293T on Day 0, Day 6, Day 12, Day 18 by cell number counting. (**G, H**) Cell morphology change upon Dox (0.5 ug/ml) induced LSD1 Knockdown for 96 hours in pLenti6.3 V5-shRNA1896/1970 infected THP-1, Molm13 and Jurkat. (**I**) Colony formation analysis of Dox (0.5 ug/ml) induced LSD1-shRNA Knockdown effects in pLenti6.3 V5-shRNA1896 infected THP-1, Molm13 and Jurkat.

Previous reports demonstrated that shRNA mediated LSD1 knockdown worked well in a variety of cells [36–40]. To validate the LSD1 knockdown efficiency in leukemia cells , we chose two of those shRNA sequences to establish eight cell lines stably expressing inducible LSD1-shRNA1896 and shRNA1970, including THP-1-sh1896/1970, Molm13-sh1896/1970, Jurkat-sh1896/1970 and HEK293T-sh1896/1970 which are maintained in RPMI1640 or DMEM with 10%FBS and Puromycin (2 ug/ml). With Doxycycline treatment (0.5 ug/ml for 4 days), both protein and mRNA levels of LSD1 decreased dramatically and there were no obvious changes on β-actin level in all cell lines (Figure 1A and 1B). After Doxycycline induction, proliferation rate of the AML cell lines (Molm13-sh1896/1970 and THP-1-sh1896/1970) decreased significantly, with ∼96.8% and ∼79.8% reduction in cell numbers observed at day 18, respectively (Figure 1C). In contrast, growth rates of the ALL cell line Jurkat-sh1896/1970, the non-leukemia cell line HEK293T-sh1896/1970 and the parental THP-1 cell line remained unchanged (Figure 1D–1E and Supplementary Figure 1A). In addition, significant morphology change for the THP-1-sh1896/1970 and Molm13-sh1896/1970 cells was observed. Cells became multi-tentacle-shaped after Doxycycline treatment (Figure 1F–1G and Supplementary Figure 1B) suggesting changes in cell differentiation status [41–44]. In contrast, depletion of LSD1 in Jurkat-sh1896/1970 cells did not change the cell morphology significantly. We then studied the effect of LSD1 knockdown on colony formation ability of these cells. After Doxycycline treatment, the number of colonies formed in THP-1-sh1896 was reduced by over 90%. Under the same condition, the clonogenicity of Molm13-sh1896 was almost completely abrogated (Figure 1H). In contrast, there were no obvious changes in colony numbers formed in Jurkat-sh1896 upon LSD1 knockdown (Figure 1H). In summary, LSD1 depletion in human AML cells THP-1 and Molm13 leads to remarkable morphology changes and significant reductions in clonogenicity and proliferation rate.

### Knockdown of LSD1 up-regulates expression levels of CD11b and CD86 in THP-1 and Molm13

**Figure 2:**
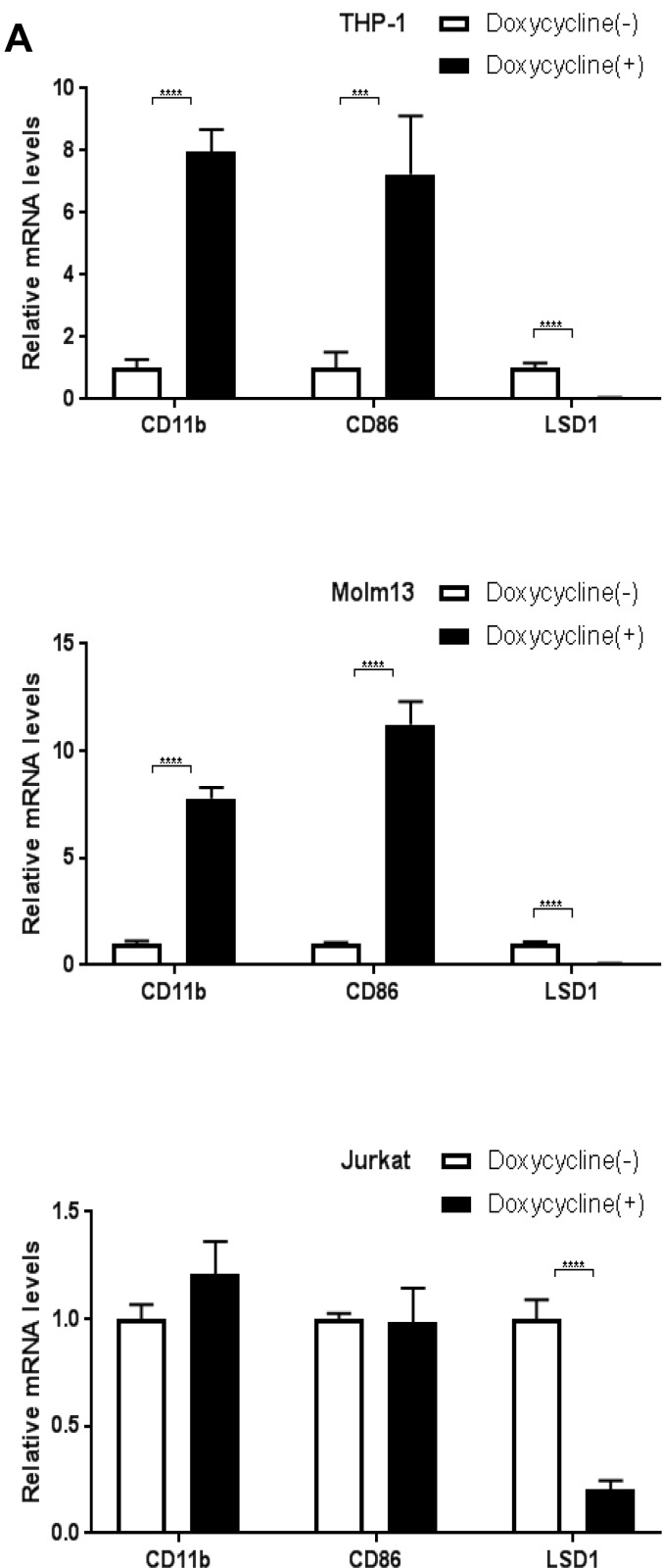

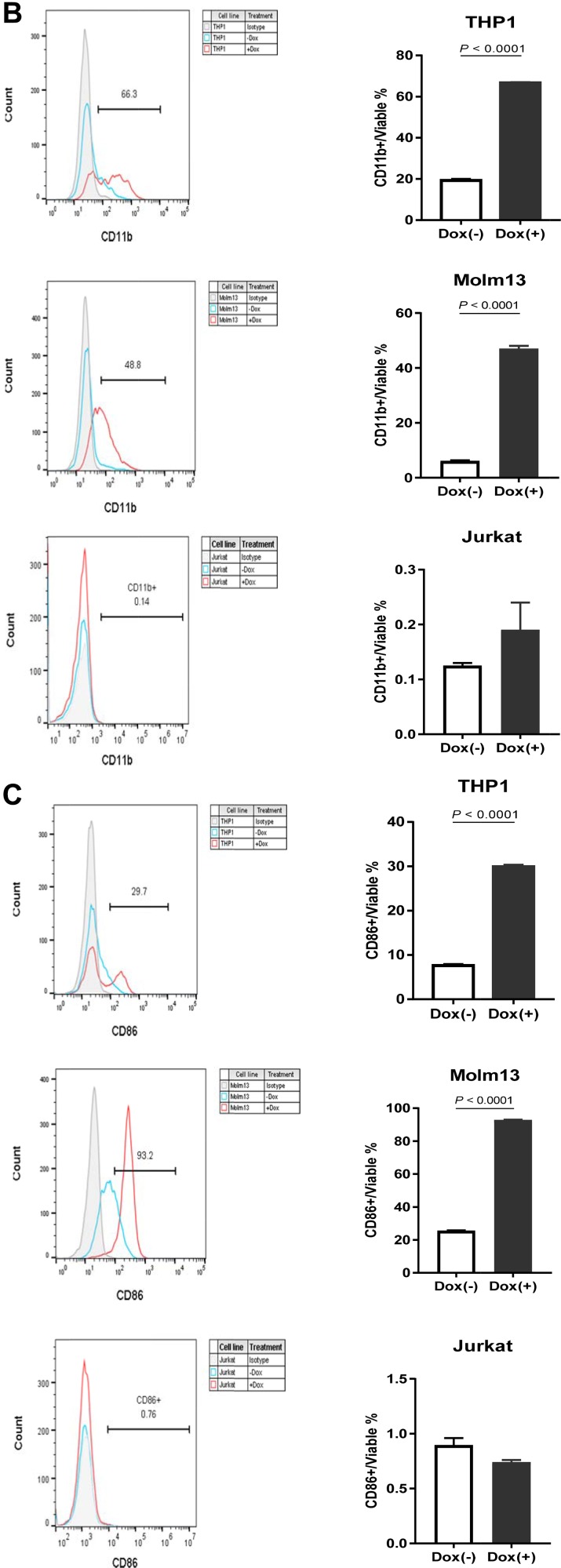
Knockdown of LSD1 upregulates CD11b and CD86 expression in AML cell lines (**A**) RT-PCR analysis of CD11b and CD86 mRNA levels upon LSD1 knockdown in THP-1, Molm13 and Jurkat. (**B**) FACS analysis of CD11b surface expression levels upon LSD1 knockdown in THP-1, Molm13 and Jurkat. (**C**) FACS analysis of CD86 surface expression levels upon LSD1 knockdown in THP-1, Molm13 and Jurkat. 7-AAD and fixable viability dye as the indicator of cell viability.

LSD1 inhibits leukemia stem cell self-renewal and induces their myeloid differentiation [1]. Literature search for the LSD1 related publications and micro-array mRNA data from the Cancer Genome Anatomy Project database (http://cgap.nci.nih.gov/Microarray/GeneList) in several cancer cell lines collected by the National Cancer Institute revealed two myeloid differentiation markers, CD11b and CD86, are potential targeted genes of LSD1 in AML [1, 2, 27, 35, 45, 46]. Based on our observation that LSD1 depletion induced cell morphology changes, we thus compared the CD11b and CD86 mRNA levels by RT-PCR and protein levels by FACS between treated and control groups for LSD1 knockdown. Our data showed that, in THP-1 and Molm13, shRNA mediated LSD1 knockdown increased mRNA levels (Figure 2A) as well as surface protein expressions (Figure 2B, 2C) of CD11b and CD86, while there was no obvious change in Jurkat. Interestingly, long term treatment with Doxycycline in THP-1 (12 days) and Molm13 (8 days) caused more significant increase on the expression of myeloid differentiation markers (CD11b and CD14) while surface expression level of CD86 went back to the control level on day 12 in THP-1 (Supplementary Figure 2A–2G). This may reflect the heterogeneity of AML with MLL-AF9 mutation and potentially different roles of CD11b and CD86 playing during different stages of myeloid differentiation. Nevertheless, our results suggest that CD11b and CD86 expression are controlled by LSD1 in AML and likely directly mediate its function in regulating myeloid differentiation [47–50].

### Knockdown of LSD1 up-regulates H3K4 methylation on the promoter regions of myeloid differentiation markers

**Figure 3:**
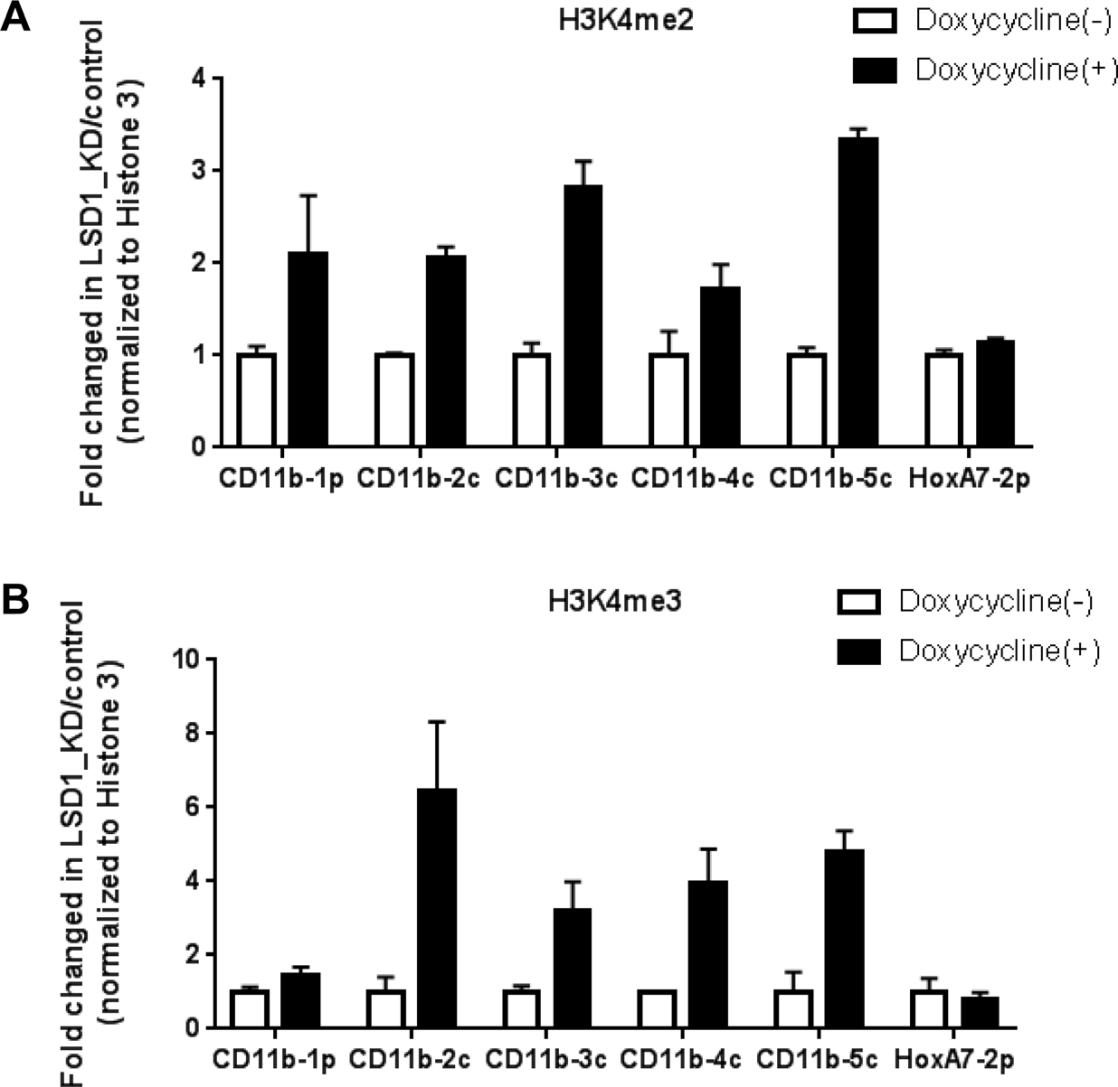

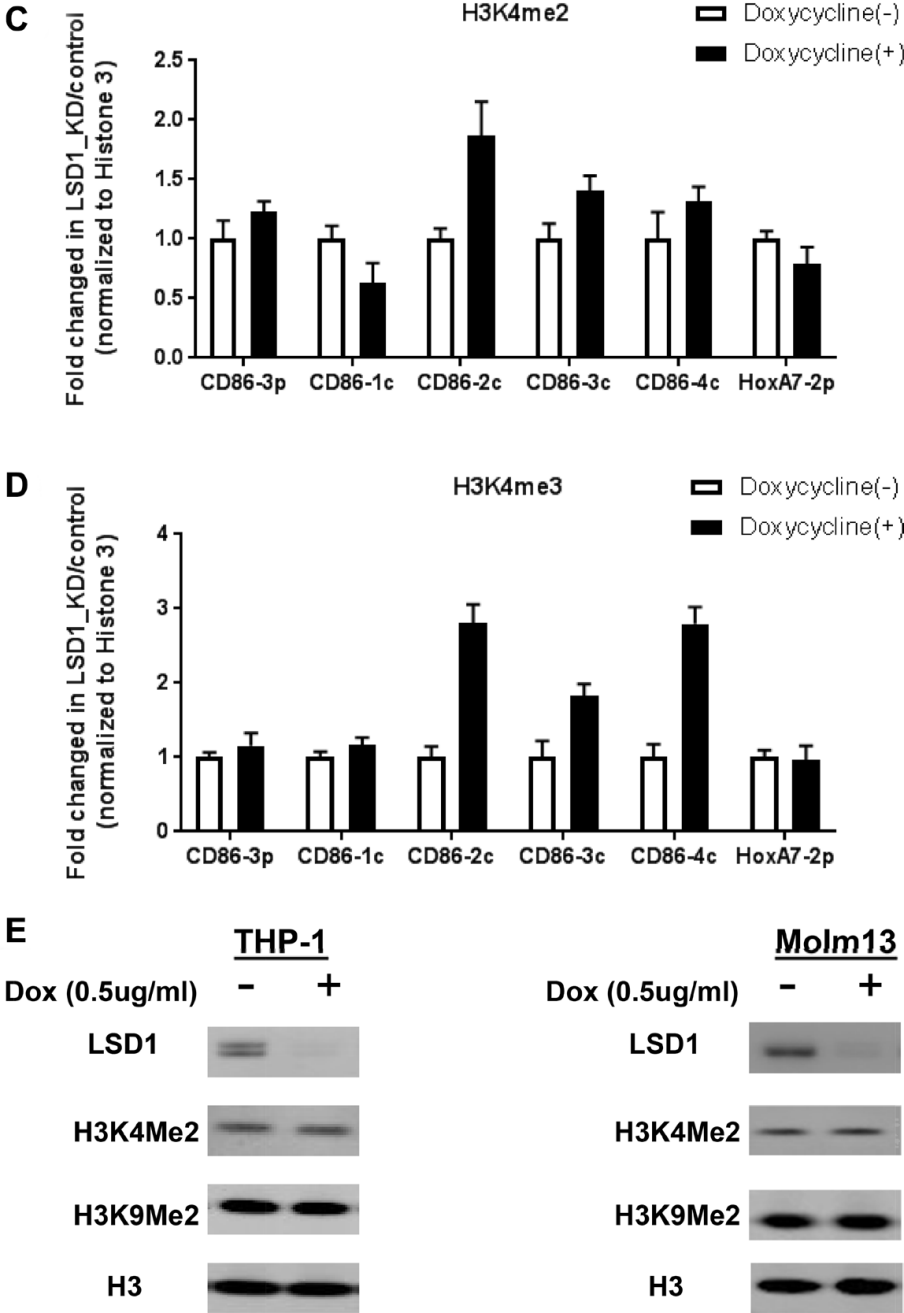
Knockdown of LSD1 increases H3K4me2 levels on the promoter regions of CD11b and CD86 in THP-1 (**A**) ChIP-qPCR analysis of H3K4me2 levels on the promoter regions of CD11b in THP-1 with Dox induced LSD1 Knockdown for 96 hours. (**B**) ChIP-qPCR analysis of H3K4Me3 levels on the promoter regions of CD11b in THP-1 with Dox induced LSD1 Knockdown for 96 hours. (**C**) ChIP-qPCR analysis of H3K4me2 levels on the promoter regions of CD86 in THP-1 with Dox induced LSD1 Knockdown for 96 hours. (**D**) ChIP-qPCR analysis of H3K4me3 levels on the promoter regions of CD86 in THP-1 with Dox induced LSD1 Knockdown for 4 days. (**E**) Western blotting analysis of global H3K4me2 and H3K9me2 levels in THP-1 and Molm13 with Dox induced LSD1 Knockdown for 96 hours. Five pairs of primers (CD11b-1p, 2c, 3c, 4c, 5c) were designed for the promoter proximal region of CD11b, five pairs of primers (CD86-3p, 1c, 2c, 3c, 4c) were designed for the promoter proximal region of CD86, with HoxA7-2p primer as the assay control. All the data are normalized to Histone 3. H3K4me2: histone 3 lysine 4 di-methylation, H3K4me3: histone 3 lysine 4 tri-methylation

LSD1 is a known H3K4 demethylase [1, 2, 4, 28, 51]. To further understand mechanistically how LSD1 regulates gene expressions of CD11b and CD86, we went on to examine whether LSD1 inhibition affects histone methylation on their promoter regions. ChIP-qPCR experiments were performed in THP-1-sh1896 and Molm13-sh1896 cells. Our results showed that H3K4me2 and H3K4me3 methylation levels were increased by 2∼3 fold and 3∼6 folds respectively at the promoter regions of CD11b and CD86 upon LSD1 knockdown, while H3K4me1 methylation levels did not show any clear trend in changes (Figure 3A–3D and Supplementary Figure 3A 3C). To further confirm these effects, we treated Molm13 with a known LSD1 inhibitor (Compound X) which has been shown to exhibit potent and concentration-dependent inhibition of the LSD1 enzymatic activity with an IC50 of 500 nM [40]. Consistent with shRNA mediated LSD1 knockdown, Compound X treatment in Molm13 cells led to strong increase in H3K4me2 and H3K4me3 levels on the promoter regions of CD11b and CD86 (Supplementary Figure 3E–3G). We also examined H3K9 methylations on CD11b and CD86 promoter regions and did not observe any obvious changes (Supplementary Figure 3B, 3D, 3H). These results together confirmed the demethylase activity of LSD1 on H3K4 dimethyl mark, and demonstrated that LSD1 indeed regulates H3K4me2, but not H3K4me1 on the promoters of CD11b and CD86 in AML cells. H3K4me3 level was also increased upon LSD1 inhibition, likely due to the accumulation of H3K4me2 and subsequent conversion by H3K4 methyltransferases. H3K9 is also been reported to be de-methylated by LSD1 in certain cellular context and plays opposite roles in H3K4 demethylation in activating or repressing gene transcription [26]. Our results suggest that this methyl-mark appears not to be affected by LSD1 inhibition at the promoters of CD11b and CD86 genes.

### Global H3K4 methylation level remains unchanged upon knockdown of LSD1

To understand whether LSD1 controls the global H3K4 methylation the LSD1, THP-1-sh1896 and Molm13-sh1896 were treated with Doxycycline (0.5 ug/ml) for 72 hours to induce LSD1 knockdown. Cell lysates were analyzed by western blotting to monitor changes in global H3K4 methylation. We found that global H3K4me2 methylation level remains unchanged after Doxycycline treatment in THP-1-sh1896 and Molm13-sh1896 cells (inducible LSD1 knockdown cell line) (Figure 3E). Concordantly, there was no significant change on global H3K4me2 level after pharmacological inhibition with a LSD1 specific inhibitor, the Trans-N-((2-methoxypyridin-3-yl) methyl)-2-phenylcyclopropan-1-amine [1, 40], suggesting that LSD1 regulates histone H3K4 modifications in a gene-specific manner.

### Knockdown of LSD1 significantly blocks tumor growth *in vivo*

**Figure 4:**
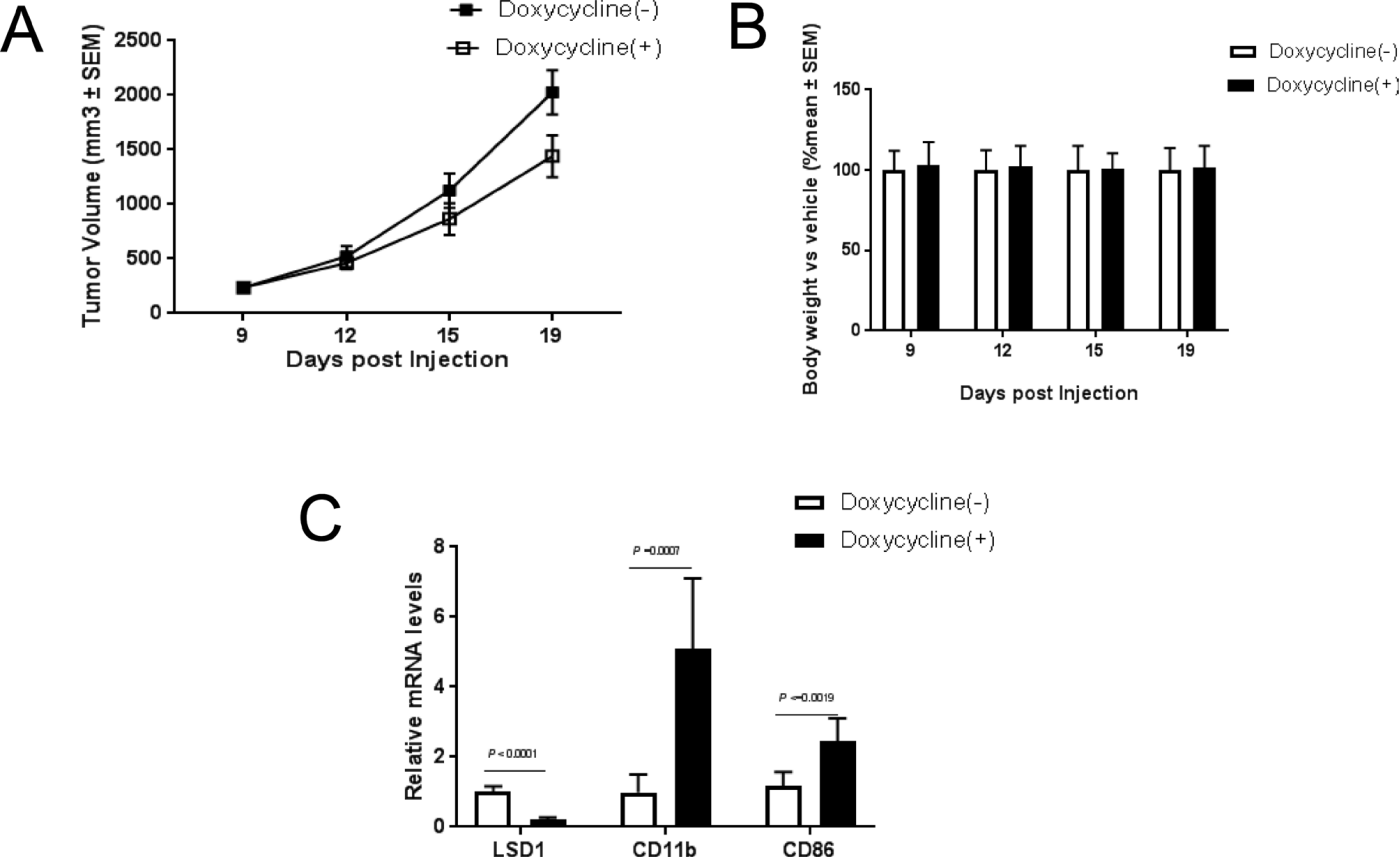
Knockdown of LSD1 inhibits tumor growth *in vivo* (**A**) Tumor growth analysis in Balb/c mouse THP-1 shRNA1896 xenograft models. Final tumor volume was compared in tumor-bearing animals receiving water contained Vehicle (10 ml/kg) and Dox (2 mg/ml) with 1% sucrose. (**B**) Body weight change analysis in Balb/c mouse THP-1 shRNA1896 xenograft models. Final body weight was compared in tumor-bearing animals receiving water contained Vehicle (10 ml/kg) and Dox (2 mg/ml) with 1% sucrose. (**C**) CD11b, CD86 and LSD1 mRNA analysis by RT-PCR in Balb/c mouse THP-1 shRNA1896 xenograft model.

To understand whether the *in vitro* effects of LSD1 knockdown could be translated into *in vivo* settings, we established a THP-1-sh1896 xenograft model as described in the material and methods. Treatment with Doxycycline significantly inhibited tumor growth with a T/C ratio of 67% (Figure 4A) and caused a dramatic increase in mRNA level of CD11b (Figure 4C). Since previous studies reported that the canonical MLL-AF9 target genes HOXA9 and Meis1 play important roles in leukemogenesis [52–56], we also investigated the expression levels of Mesi1, HoxA9 and HoxA10. Interestingly, the expression level of Meis1 was significantly reduced upon LSD1 knockdown while HoxA9 and HoxA10 was not altered (Supplementary Figure 4A). In summary, our observations indicate that LSD1 knockdown showed robust antitumor efficacy in the THP-1 xenograft tumor model, which is likely mediated through promoting myeloid differentiation and attenuating proliferation *in vivo*.

## DISCUSSION

MLL translocations occur most frequently in Acute Monocytic Leukemia (French-American-British classification AML-FAB-M5) with an overall incidence of ∼30% [57]. Of over 80 different translocation partner genes, the translocation t (9; 11) (p22; q23) leading to the leukemogenic fusion gene MLL-AF9 is a frequent translocation in infant acute myeloid leukemia (∼50%) and especially associated with infant acute monocytic leukemia with MLL translocation (AML-FAB-M5, ∼70%) [58–61]. MLL and AF9 wild type proteins play essential roles in embryogenesis and hematopoiesis. They also are parts of protein complexes complexes leading to transcriptional initiation (MLL) and elongation (AF9) [62–67]. MLL-AF9 fusion protein is believed to recruit LSD1 to protein complexes and allow LSD1 to demethylase H3K4me2 in the specific promoter regions of target genes, leading to abnormal transcriptional initiation and elongation of these genes [68, 69].

In this study, we investigated the role of LSD1 in regulating proliferation and differentiation in human MLL-AF9 translocated monocytic leukemia cells. THP-1 and Molm13 cell lines were chosen because THP-1 is the only one AML cell line from a characteristic infant monocytic AML M5 leukemia patient, and Molm13 is the classical cell line established from the peripheral blood of a nine years old boy patient with relapsed acute monocytic AML M5a leukemia. Together, both cell lines have been greatly confirmed to be the most common cellular models for studying monocytic differentiation and leukemogenesis [62, 70].

Previous studies demonstrated that genetic knockdown of MLL-AF9 induced the expression of pro-apoptotic regulators (EBPB, DUSP1, HIPK2, TGFBR1) while reducing the expression of anti-apoptotic regulators (MEF2C, SOCS2, SOX4), leading to impaired proliferation and apoptosis in THP-1 and Molm13 [62, 71]. In line with this, we found that knockdown of LSD1 dramatically attenuates the cellular proliferation and colony formation ability of THP-1 (MLL-AF9, M5), and Molm13 (MLL-AF9, M5a), with no activity against proliferation of Jurkat (MLL-WT, wide type of MLL) (Supplementary Figure 1D, 1E, 1F). We further showed the anti-leukemogenesis activity of LSD1 knockdown is associated with significant increases of two myeloid differentiation markers, CD11b and CD86, suggesting that LSD1 inhibition not only leads to reduced proliferation but also induces differentiation of the MLL-AF9 monocytic cells. Consistent with our results, Roscher's group reported that genetic knockdown of MLL-AF9 induced the expression of moncytic mature markers (CD14, CEBPB, EGR2) and reduced the expression of immature monocytic lineage markers (ELANE and CTSG) in THP-1 [62, 72–75]. Therefore, MLL-AF9 and LSD1 might regulate monocyte differentiation with a shared mechanism. It was reported that LSD1 co-localizes with genes bound by MLL-AF9 and demethylases H3K4me2 on the specific promoter regions of these genes, with little effects on the global H3K4me2 level [1]. Our data indicated that LSD1 inhibition indeed induces expression of CD11b and CD86 through increasing H3K4me2 levels on the proximal promoter region of those two genes, although global H3K4me2 level remains unaltered, suggesting that the pro-differentiation effect of LSD1 inhibition depends on its histone demethylase activity on these specific target genes (CD11b and CD86). Consistent with our findings, others groups have reported that pharmacological inhibition of LSD1 increases H3K4 or H3K9 methylation in these genes [1, 2, 20, 25]. Interestingly, Feng and Fiskus’s groups reported that pharmacological inhibition of LSD1 globally increases H3K4 methylation in some specific AML cells [4, 27], which suggests it is a cell type dependent pattern. Importantly, knockdown of LSD1 demonstrates strong anti-tumor effects in THP-1 (MLL-AF9) xenograft model, accompanied with increased CD11b and CD86 expression. In Addition, we also noticed that two very recent studies showing that pharmacological inhibition of LSD1 has significant anti-tumor effects in MV4-11(MLL-AF4) systemic model and Kasumi-1(RUNX1(AML1)/CBFA2T1(ETO)) xenograft model [4, 27, 32]. It suggests that genetic inhibition of LSD1 could also harbor a broader anti-tumor spectrum in different MLL subtypes of AML. Together, targeting LSD1 represents a potential therapeutic strategy against MLL-AF9 translocated acute monocytic leukemia (FAB-M5) in infants and children. Therefore, infants and children with MLL-AF9 monocytic leukemia might be a suitable patient group for clinical trials of LSD1 inhibitors. Multiple oral LSD1 inhibitors have already been undergoing phase I or IIa clinical trial in patients with AML (https://clinicaltrials.gov/; ORY-1001 in phase I/IIa study; GSK2879552 in phase I study). In addition, we noticed that Lynch's group reported that physical association of LSD1 with transcription factors such as GFI1 is essential to maintain the differentiation block in AML and tranylcypromine-derivative inhibitors target this novel scaffolding function of LSD1, rather than its histone demethylase activity, to promote differentiation of AML cells [76]. It suggests that LSD1 might regulate myeloid differentiation and oncogenic proliferation on at least three dimensions: the first one is histone modification, such as H3K4 methylation; the second is non-histone protein methylation, such as p53-K372 methylation; and the last one is non-enzymatic scaffolding function, such as LSD1-GFI1 interaction in MLL-AF9 translocated AML, indicating the complexity of LSD1 function in the different cell contexts. Meanwhile, several evidences showed that expression of CD11b and CD86 can be significantly induced after treatment with LSD1 inhibitors in THP-1, Molm13, MV4-11 and other AML cells, which is highly consistent with our results [1, 4, 51, 76–79]. Given that all unclear complexity of LSD1 in AML, the combination therapy could be the attentive therapy strategy in practice. Recently, several groups reported that addition of LSD1 inhibitors to other molecular entities could be a promising therapy against AML, such as DNMT inhibitor, pan-HDAC inhibitor, Dot1L inhibitor and ATRA (all trans-retinoid acid) [2, 4, 80, 81].

**Figure 5:**
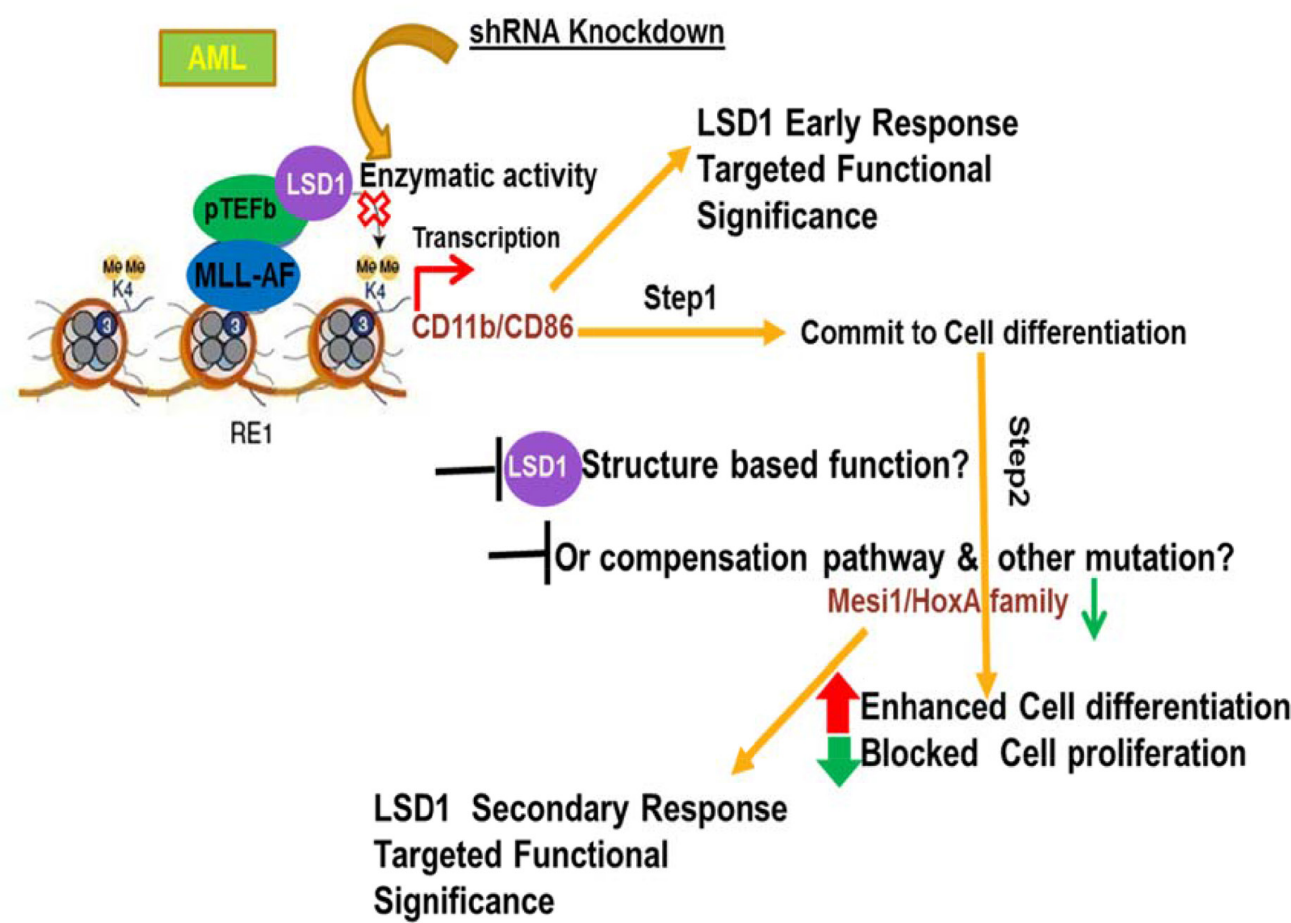
Proposed model of mechanism demonstrating that LSD1 regulates myeloid differentiation and oncogenic proliferation in MLL-AF9 AML cells In MLL-AF9 AML, cellular differentiation may be divided into two stages that are differentiated controlled by LSD1 knockdown. In the first step, LSD1 inhibition quickly leads to increased CD11b and CD86 levels, which results in commitment to myeloid differentiation of cells. In the second step, with the long term sustained treatment of LSD1 knockdown, while CD11b, CD86 are continually upregulated, oncogenic genes, such as HoxA9 and Meis1, are gradually repressed in cells. As a result, cells enter into late differentiation stage and cell proliferation ability begun to be dramatically reduced.

Taken together, we propose that the anti-AML activity of LSD1 inhibition could be attributed to its function in promoting myeloid differentiation and inhibition of oncogenic proliferation through its demethylation activity (Figure 5). Our findings indicate that therapies targeting demethylase LSD1 may be a potential strategy to treat acute monocytic leukemia with MLL-AF9 translocation in infants and children.

## MATERIALS AND METHODS

### Cell lines and reagents

SDS, Glycerol, Tris-HCl, Iodonitro-tetrezolium chloride, Ethanol, Doxycycline (Dox) was purchased from Sigma (St. Louis, MO, USA). All AML cell lines, THP-1, Molm13, Jurkat, HEK293T purchased from ATCC were maintained in RPMI1640 or DMEM medium supplemented with 10% fetal bovine serum (Cat.No.10010-147, Gibco, Life technology) and 1% penicillin/streptomycin (Cat. No. 15140-163, Gibco, Life technology) and grown at 37°C in 5% CO2 atmosphere.

### Generation of LSD1-shRNA lentiviral particle

Human LSD1 shRNA-1896 (NM-015013: CCGGCCACGAGTCAAACCTTTATTTCTCGAGAAATAAAGGTTTGACTCGTGGTTTTTG) and shRNA-1970 (NM-015013: CCGGGCCTAGACATTAAACTGAATACTCGAGTATTCAGTTTAATGTCTAGGCTTTTTG) were synthesized and cloned into the pLKO.1 vector with the Puromycin resistant gene according to the manufacturer’s instructions (Sigma-Aldrich, St. Louis, MO, USA). The 293FT cells were transfected with pLKO.1-LSD1 shRNA-1896 using the Vira Power^™^ HiPerform^™^ (Life Technology, invitrogen, CA, USA). After overnight, medium was replaced with fresh culture medium. After an additional 72 hours of culture, media were collected and filtered through a 0.45 um syringe filter to remove floating cells, and the aliquot supernatant was stocked in –80°C freezer and used for infection in future.

### Infection of leukemia cells with lentivirus

Cells (8*10E5/mL) were incubated in 6-well plates (Corning) with 1000 uL RPMI-1640 or DMEM supplemented with 10% FBS and 1000 uL of lentiviral supernatant. Polybrene (8 ng/mL) was added to the viral supernatant at a ratio of 1:1,000 (vol/vol). And centrifuged the 6-well plate with 300 g at room temperature (25°C) for 90 min, then cultured at 37°C in 5% CO2 atmosphere for overnight. On the following day, cells were harvested and cultured in RPMI-1640 supplemented with 10 % FBS and Puromycin (1–2 ug/ml) at least for 3 days.

### Cell number counting assay

All cells are seeded at 1*10E5/5*10E4 with different dose of compounds in 12-well or 24 well plate on day 0 were incubated in RPMI1640 or DMEM medium supplemented with 10% fetal bovine serum and 1% penicillin/streptomycin and grown at 37°C in 5% CO2 atmosphere. Refreshed the medium and counted the cell numbers as the protocol by Vi-cell cell viability analyzer system (Beckman Coulter, CA, USA) on different days. All data were finally analyzed by Prism5-GraphPAD.

### Colony formation assay

AML cells seeded at 300 per well in a 48-well plate were incubated in human methylcellulose base media with compounds of the required dose for 10–21 days according to the manufacture’s recommendation (Cat.no.HSC002, R&D systems, Minneapolis, MN, USA). Cells were fixed and stained with Iodonitrotetrezolium chloride (8 mg/ml dissolved in ethanol) for above 2 hours, quantitated by Quantity One software in Gel-DocTM XR+ with Image LabTM software system (Bio-Rad, Hercules, CA, USA).

### Western blot

Cells were treated as required and washed twice with ice-cold PBS, lysed in SDS lysis buffer (2% SDS, 10% Glycerol, 0.625 M Tris-HCl, ph6.8), applied to western blot analysis. 1–2 ug of total proteins were loaded on 4–12% NuPAGE Bis–Tris gels (Life technology) and transferred onto NC or PVDF membranes by iBLOT transferring system (Life technology). Primary antibodies against Histone H3 was purchased from Sigma-Aldrich (St. Louis, MO, USA) and H3K4me1 (Cat.no. ab8895) and H3K9me2 (Cat.no. ab1220) was purchased from ABcam (Cambridge, MA, USA) H3K4me2 (Cat.no. 07-059) were purchased from Millipore (Billerica, MA, USA), LSD1 (CST#2139) was purchased from Cell Signaling Technology (Danvers, MA, USA). β-Actin (Cat.no. A1978) was purchased from Sigma-Aldrich (St. Louis, MO,USA). Secondary antibodies again Rabbit (Cat.No.926-68073) and Mouse (Cat.No.926-32212) were purchased from Li-COR (Lincoln, NE, USA). The Odyssey Infrared Detection System (LI-COR Biosciences, Lincoln, NE, USA) was used to quantify relative amounts of proteins.

### RNA isolation and quantitative RT-PCR

Total RNA was prepared by using RNeasy mini kit on Qubic workstation according to manufacturer’s instruction (QIAGEN, Madison, WI, USA). A total of 0.5 ug RNA was treated with RNase H and then reverse transcribed by using the Superscript III First strand synthesis super-mix for RT-PCR, following manufacturer’s suggestion (Cat.no.11752-250, Life Technologies, and Grand Island, NY, USA). The quantity of cDNA was determined by qPCR analysis using Power SYBER green PCR master mix(Cat.no.11752-250, Life Technologies, Grand Island, NY,USA) in ABI 7900HT Fast Real –time PCR System (Life Technologies, Grand Island, NY,USA). Primer sequences and conditions for qPCR are available upon request.

### Analysis of myeloid differentiation and apoptosis

Cells were induced LSD1 knockdown by treatment with Dox at 0.5 ug/ml for four days before analysis. Alexa Fluor^®^ 488 mouse anti-human CD14 monoclonal antibody (Clon M5E2, Cat.no.#301817, BioLegend), PE-conjugated mouse anti-human CD11b monoclonal antibody (Clone ICRF44, Cat.no.#555388, BD Pharmingen), APC-conjugated mouse anti-human CD86 monoclonal antibody (Clone 2331, Cat.no.#555660, BD Pharmingen) and Alexa Fluor^®^ 647 mouse anti-human CD68 Antibody (Clone Y1/82A, Cat.no. #333819, BioLegend) are used for FACS staining at 1:200 dilution. FACS samples were acquired using Guava EasyCyte 8HT flow cytometer (EMD Millipore) and CytoFlex S flow cytometer (Beckman Coulter). Cell viability was also examined by FACS analysis with 7-AAD or Fixable Viability dye (Thermo Fisher Scientific) according to the manufacturer‘s instructions. Results were then analyzed with FlowJo V10 software (TreeStar Inc.).

### Chromatin Immunoprecipitation (ChIP)

Cells were treated with Dox at 0.5 ug/ml for four days prior to analysis. Chromatin shearing was performed using Qsonica Sonicators Q700 (Qsonica, Newton, CT) to 100–300 base pairs, according to manufacturer’s instructions. ChIP was performed using the Chromatin IP(ChIP) Assay Kit (Millipore)according to standard protocols and the supplier’s directions with ChIP-grade antibodies : anti-H3K4me1(Abcam, #ab8895), anti-H3 total(Cell signaling, #2650) anti-H3K4me2(Millipore, #05-790), anti-H3K4me3(Cell signaling, #9727) and anti-H3K9me2(Abcam, #ab1220).We used normal mouse IgG (Millipore, #12-371) and normal rabbit IgG(Cell signaling, #2729) as a negative control.

### THP-1-sh1896 xenograft tumor model

The experiments were conducted on female BALB/c nude mice (from Shanghai SLAC Laboratory animal Co., LTD) aged 4–6 weeks old, weighing approximately 18–22g. The animals were housed in the specific pathogen free animal facility in accordance with the guide for care and use of laboratory animals and the regulations of the institutional animal care and use committee. Each mouse was be inoculated subcutaneously at the right flank with the 5 × 10^6^ of Thp1 sh1896 Human Leukemia cell in PBS with 50% matrigel for the tumor development. Measure the tumor volume and body weight twice every week. Tumor sizes will be measured twice weekly in two dimensions using a caliper, and the volume will be expressed in mm3 using the formula: V = 0.5a × b2 where a and b are the long and short diameters of the tumor, respectively. The tumor sizes are then used for the calculations of both T-C and T/C values. T-C is calculated with T as the median time (in days) required for the treatment group tumors to reach a predetermined size (e.g., 1,000 mm3), and C is the median time (in days) for the control group tumors to reach the same size. The T/C value (in percent) is an indication of antitumor effectiveness, which is calculated using the formula: T/C% = Ti–T0/Ci–C0 * 100%, Ti is the average tumor volume of a treatment group on a given day, T0 is the average tumor volume of the corresponding treatment group on the day of treatment start, Vi is the average tumor volume of the vehicle control group on the same day with Ti, and V0 is the average tumor volume of the corresponding vehicle group on the day of treatment start. The 2.0 mg/ml Doxy + 1% or 5% sucrose was administrated through the drinking the water.

### Statistical analysis

All data are expressed as the mean ± standard deviation (SD). Statistical differences between experimental groups were determined using Student’s *t*-test. All statistical analyses were performed using Prism 5/GraphPad and SPSS 13.0. A value of *p* < 0.05 was considered statistically significant (n.s.: *p* > 0.05; _*_: 0.01 < *p* ≤ 0.05; _**_: 0.001 < *p* ≤ 0.01; _***_: 0.0001 < *p* ≤ 0.001; _****_: *p* ≤ 0.0001).

## SUPPLEMENTARY MATERIALS FIGURES


