# Supplementary material for: Upregulation of CD11b and CD86 through LSD1 inhibition promotes myeloid differentiation and suppresses cell proliferation in human monocytic leukemia cells
Source: Oncotarget. 2017 Jun 19;8(49):85085–101. doi: 10.18632/oncotarget.18564 (PMC5689595; doi:10.18632/oncotarget.18564)
Supplement: Supplementary file 1 [file oncotarget-08-85085-s001.pdf]

Upregulation of CD11b and CD86 through LSD1 inhibition promotes myeloid differentiation and suppresses cell proliferation in human monocytic leukemia cells

SUPPLEMENTARY MATERIALS

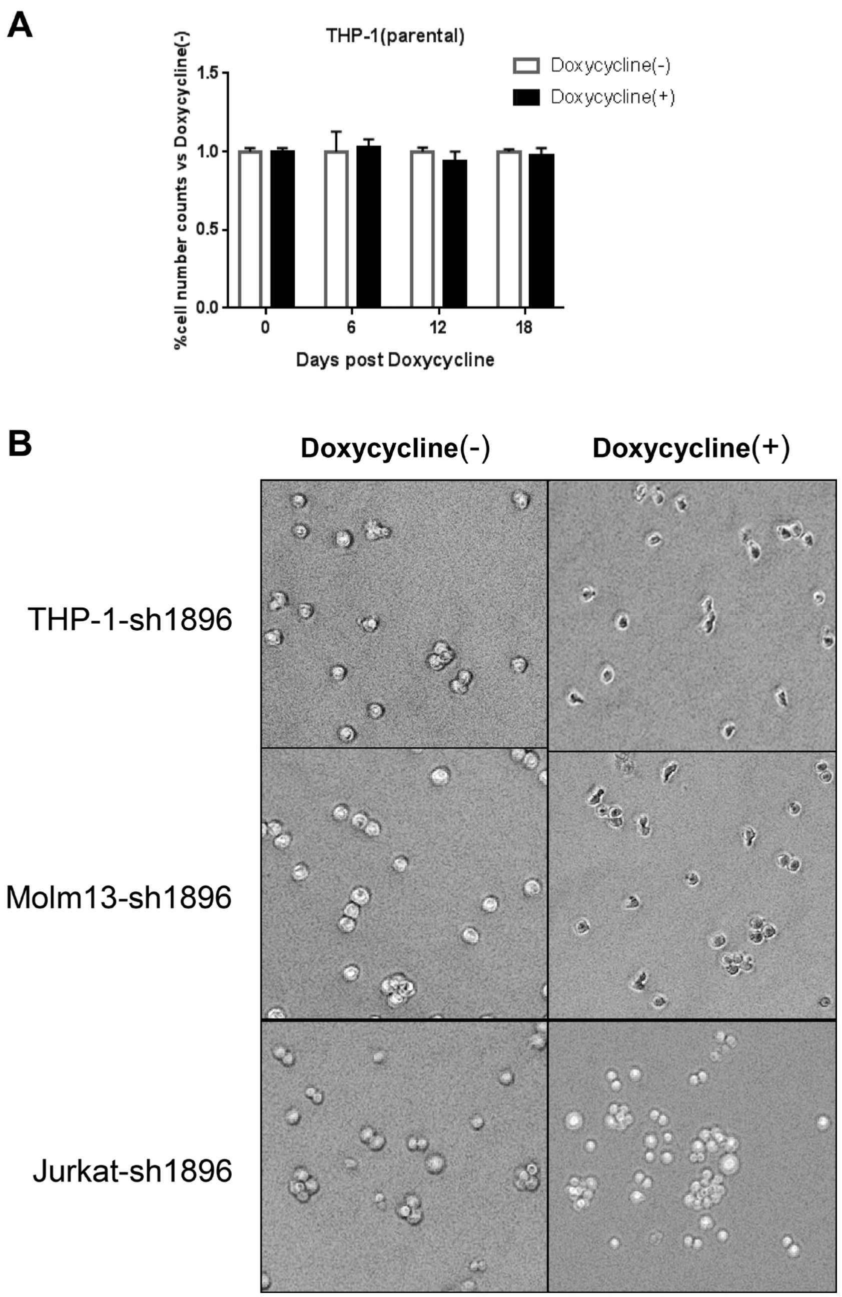

Supplementary Figure 1: Analysis of cell proliferation and morphology in THP-1, Molm13 and Jurkat with LSD1 knockdown. (**A**) Effects of Dox (0.5 ug/ml) induced LSD1 Knockdown effect on cell growth in THP-1(parental) on Day 0, Day 6, Day 12, Day18 by cell number counting. (**B**) Cell morphology change upon Dox (0.5 ug/ml) induced LSD1 Knockdown for 96 hours in pLenti6.3 V5-shRNA1896/1970 infected THP-1, Molm13 and Jurkat at a lower cell density.

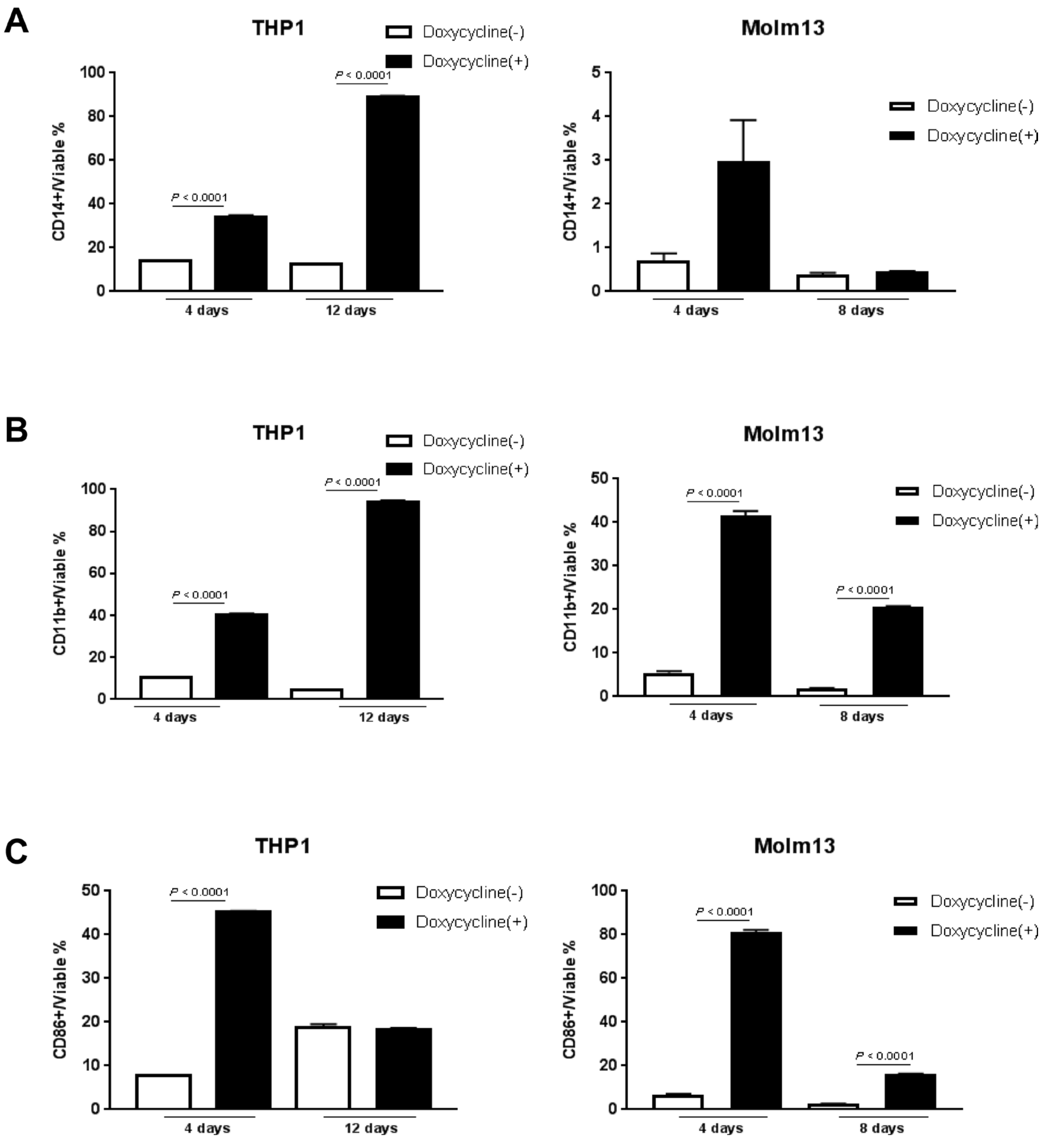
A
THP1
Doxycycline(-)
Doxycycline(+)
CD14+/Viable %
100
80
60
40
20
0
P < 0.0001
P < 0.0001
4 days
12 days
Molm13
CD14+/Viable %
5
4
3
2
1
0
Doxycycline(-)
Doxycycline(+)
4 days
8 days
B
THP1
Doxycycline(-)
Doxycycline(+)
CD11b+/Viable %
100
80
60
40
20
0
P < 0.0001
P < 0.0001
4 days
12 days
Molm13
CD11b+/Viable %
50
40
30
20
10
0
P < 0.0001
P < 0.0001
Doxycycline(-)
Doxycycline(+)
4 days
8 days
C
THP1
CD86+/Viable %
50
40
30
20
10
0
P < 0.0001
Doxycycline(-)
Doxycycline(+)
4 days
12 days
Molm13
CD86+/Viable %
100
80
60
40
20
0
P < 0.0001
P < 0.0001
Doxycycline(-)
Doxycycline(+)
4 days
8 days

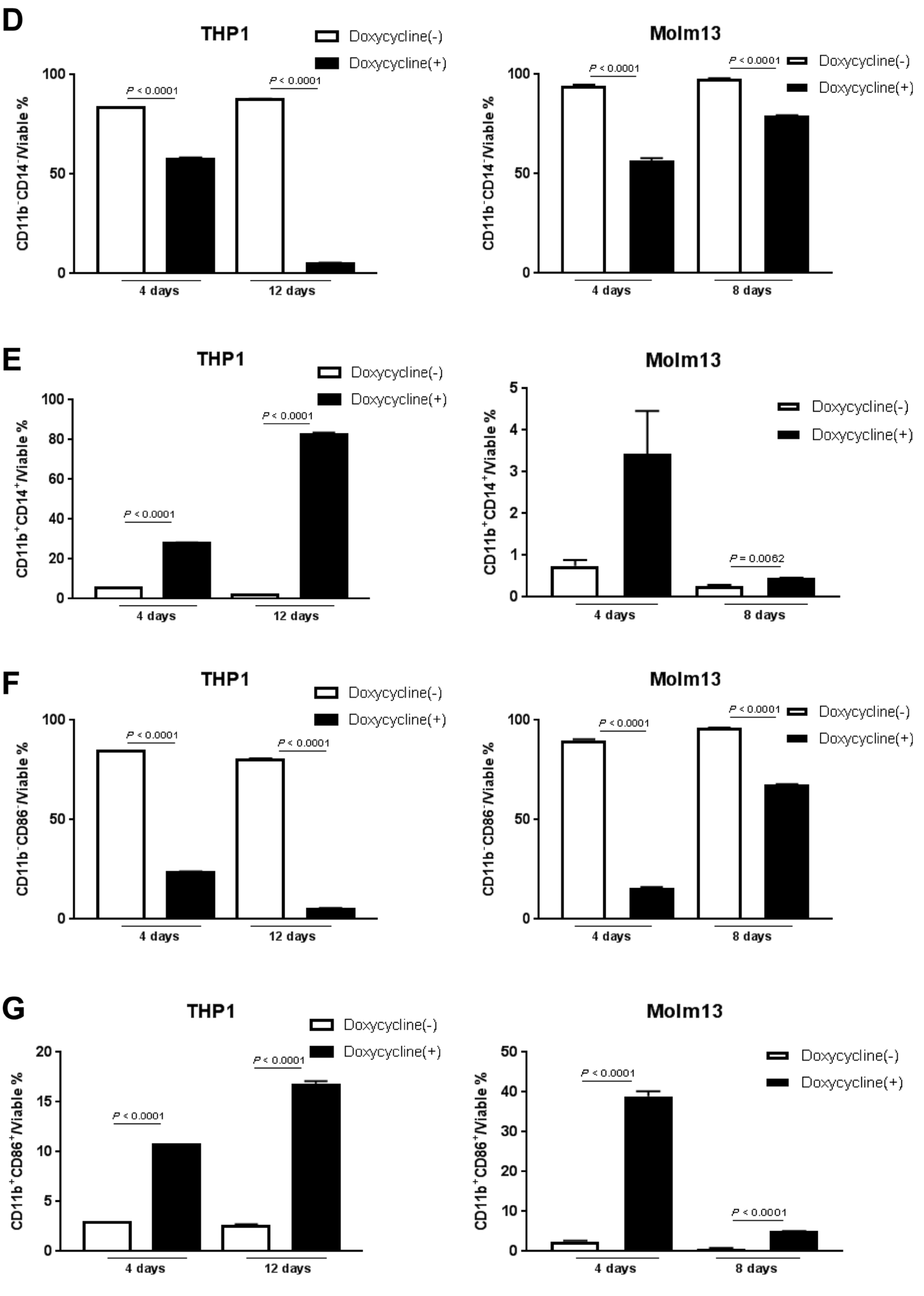

Supplementary Figure 2: FACS analysis of surface expression levels of CD14, CD11b and CD86 in THP-1 and Molm13 with long term knockdown of LSD1. (**A**–**C**) FACS analysis of Dox (0.5 ug/ml) induced LSD1-shRNA Knockdown for 4 days, 8 days (Molm13) or 12 days (THP-1) on CD14, CD11b and CD86 protein level in THP-1 and Molm13. (**D**–**G**) FACS analysis of Dox (0.5 ug/ml) induced LSD1-shRNA Knockdown for 4 days, 8 days or 12 days on CD11b$^-$CD14$^-$, CD11b$^+$CD14$^+$, CD11b$^-$CD86$^-$, CD11b$^+$CD86$^+$ protein level in THP-1 and Molm13. Use 7-AAD or fixable viability dye as the indicator of cell viability.

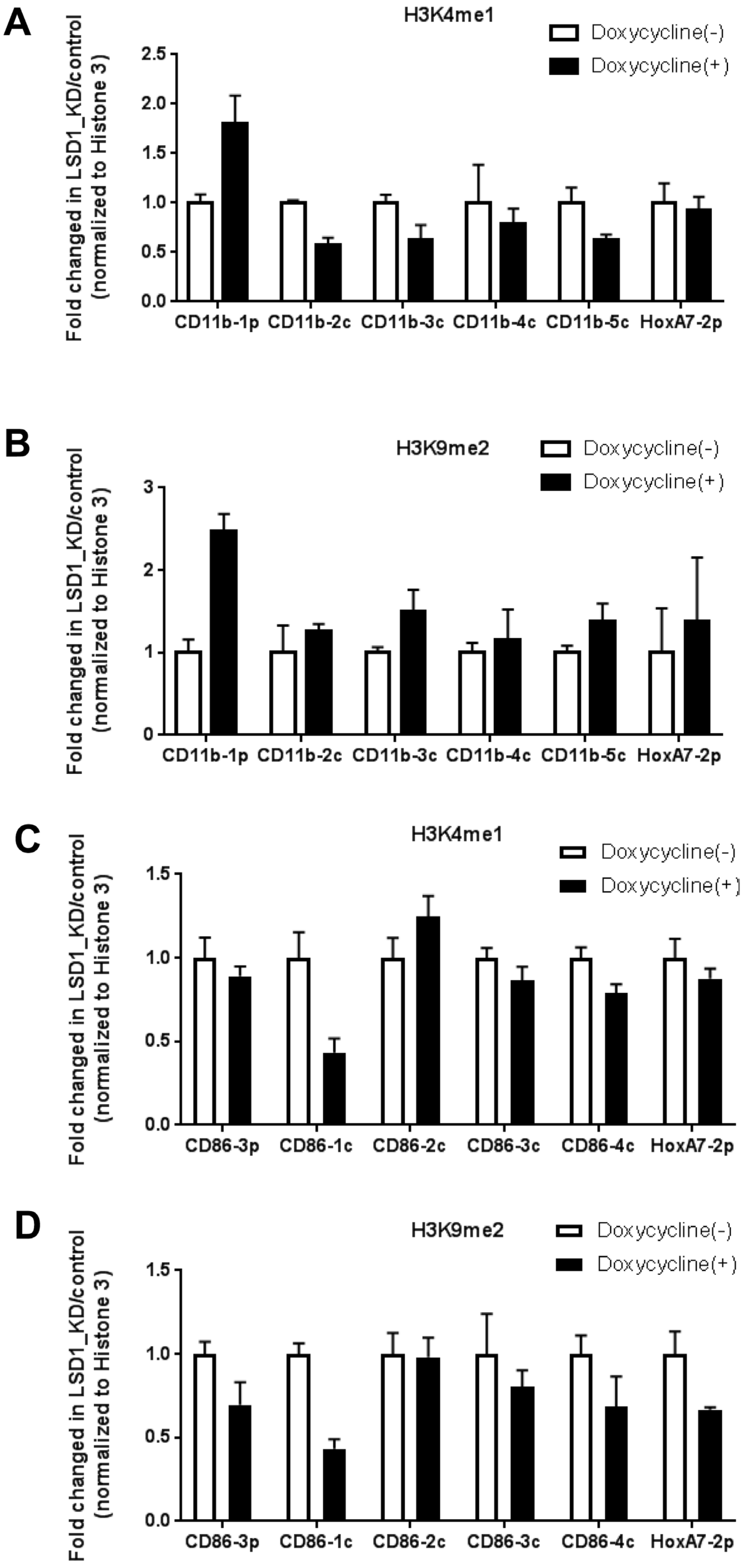

A
H3K4me1
Doxycycline(-)
Doxycycline(+)
Fold changed in LSD1_KD/control
(normalized to Histone 3)
2.5
2.0
1.5
1.0
0.5
0.0
CD11b-1p
CD11b-2c
CD11b-3c
CD11b-4c
CD11b-5c
HoxA7-2p
B
H3K9me2
Doxycycline(-)
Doxycycline(+)
Fold changed in LSD1_KD/control
(normalized to Histone 3)
3
2
1
0
CD11b-1p
CD11b-2c
CD11b-3c
CD11b-4c
CD11b-5c
HoxA7-2p
C
H3K4me1
Doxycycline(-)
Doxycycline(+)
Fold changed in LSD1_KD/control
(normalized to Histone 3)
1.5
1.0
0.5
0.0
CD86-3p
CD86-1c
CD86-2c
CD86-3c
CD86-4c
HoxA7-2p
D
H3K9me2
Doxycycline(-)
Doxycycline(+)
Fold changed in LSD1_KD/control
(normalized to Histone 3)
1.5
1.0
0.5
0.0
CD86-3p
CD86-1c
CD86-2c
CD86-3c
CD86-4c
HoxA7-2p

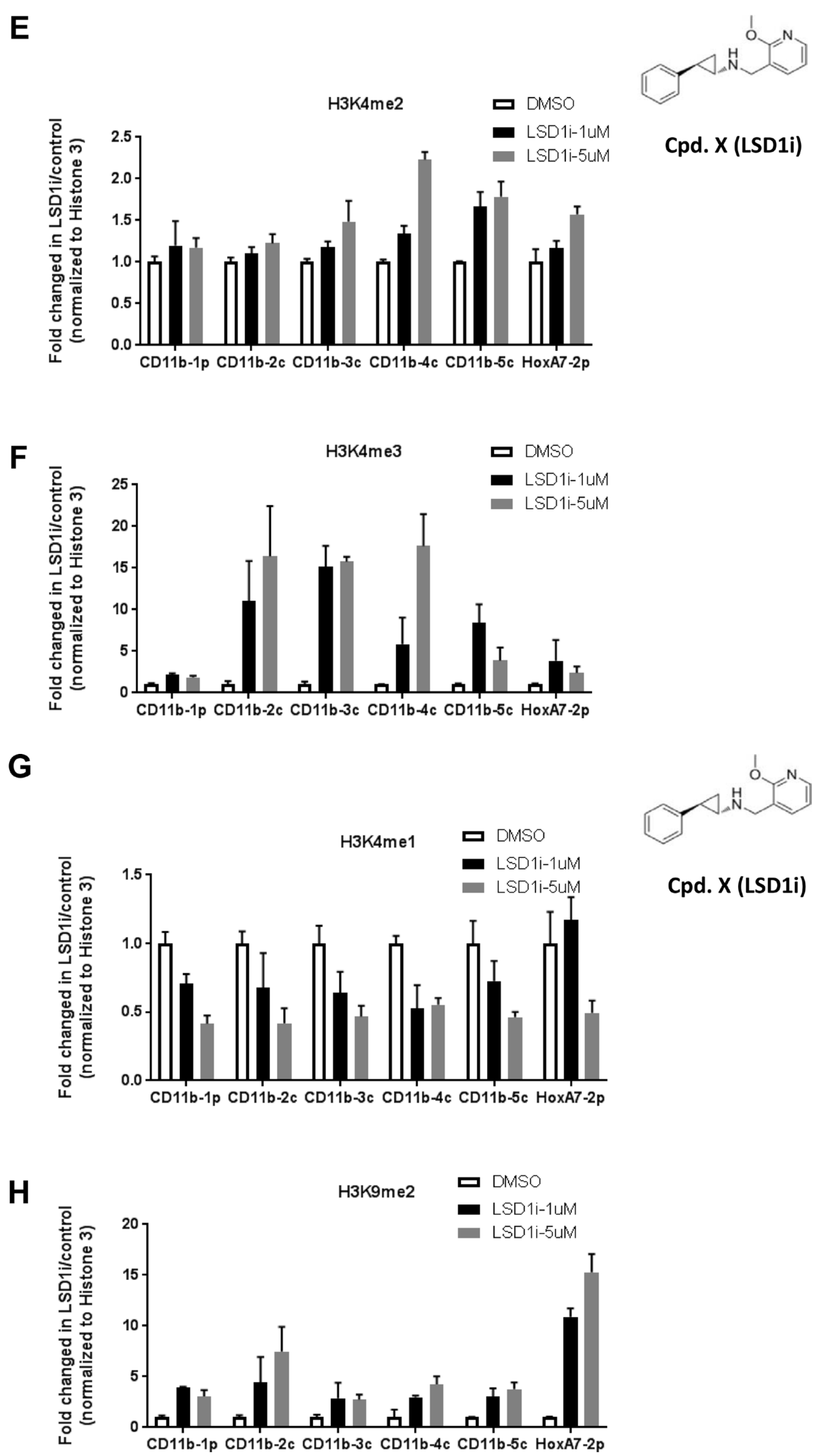

Supplementary Figure 3: ChIP-qPCR analysis of H3K4me1 and H3K4me3 levels on the promoter regions of CD11b and CD86 in THP-1 and Molm13. (**A**) ChIP-qPCR analysis of H3K4me1 levels on the promoter regions of CD11b in THP-1 with Dox induced LSD1 Knockdown for 96 hours. (**B**) ChIP-qPCR analysis of H3K9me2 levels on the promoter regions of CD11b in THP-1 with Dox induced LSD1 Knockdown for 96 hours. (**C**) ChIP-qPCR analysis of H3K4me1 levels on the promoter regions of CD86 in THP-1 with Dox induced LSD1 Knockdown for 96 hours. (**D**) ChIP-qPCR analysis of H3K4me3 levels on the promoter regions of CD86 in THP-1 with Dox induced LSD1 Knockdown for 96 hours. (**E**) ChIP-qPCR analysis of H3K4me2 levels on the promoter regions of CD11b in Molm13 treated with LSD1i for 96 hours. (**F**) ChIP-qPCR analysis of H3K4me3 levels on the promoter regions of CD11b in Molm13 treated with LSD1i for 96 hours. (**G**) ChIP-qPCR analysis of H3K4me1 levels on the promoter regions of CD11b in Molm13 treated with LSD1i for 96 hours. (**H**) ChIP-qPCR analysis of H3K9me2 levels on the promoter regions of CD11b in Molm13 treated with LSD1i for 96 hours. Five pairs of primers (CD11b-1p, 2c, 3c, 4c, 5c) were designed for the promoter regions of CD11b. Five pairs of primers (CD86-3p, 1c, 2c, 3c, 4c) were designed for the promoter regions of CD86 with HoxA7-2p primer as the assay control. All the data are normalized to Histone 3.

A

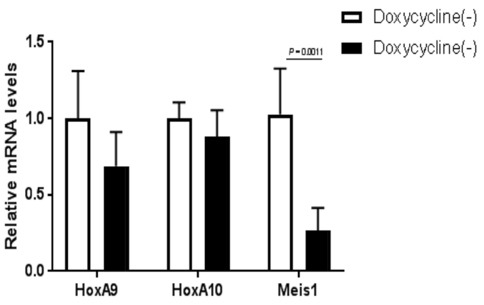

B

Treatment	Tumor response		Host response		
	T/C (%)	Mean change of tumor volume (mm^3 ± SEM)	Mean change of body weight (g ± SEM)	% change of body weight (mean ± SEM)	Survival (Survivors / total)
Vehicle(10 mL/kg ip QD)	100	1790 ± 180	4.2 ± 0.4	14.8 ± 2.2	8/8
Doxycycline(2 mg/mL in 1% Sucrose)	67	1203 ± 175 *	3.4 ± 0.6	12.7 ± 2.1	8/8

*: p <0.05 by student t-test, vs Vehicle group

C

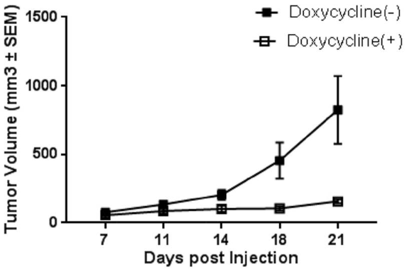

D

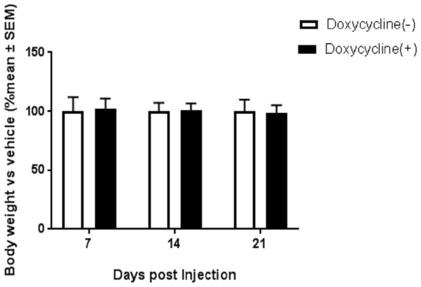

E

Treatment	Tumor response		Host response		
	T/C (%)	Mean change of tumor volume (mm^3 ± SEM)	Mean change of body weight (g ± SEM)	% change of body weight (mean ± SEM)	Survival (Survivors / total)
Vehicle(10 mL/kg ip QD)	100	1396 ± 420	1.6 ± 0.3	6.91 ± 1.27	8/8
Doxycycline(2 mg/mL in 5% Sucrose)	16	221 ± 42 *	0.8 ± 0.2	3.4 ± 0.95	7/7

*: p = 0.003 by student t-test, vs Vehicle group

Supplementary Figure 4: Knockdown of LSD1 inhibits tumor growth *in vivo*. (**A**) Meis1, HoxA9 and HoxA10 mRNA levels analysis by RT-PCR in Balb/c mouse THP-1 shRNA1896 xenograft model. (**B**) Statistical analysis of anti-tumor growth effect in Balb/c mouse THP-1 shRNA1896 xenograft model. (**C**) Tumor growth analysis in Balb/c mouse THP-1 shRNA1896 xenograft models. Final tumor volume was compared in tumor-bearing animals receiving water contained Vehicle (10 ml/kg) and Dox (2mg/ml) with 5% sucrose. (**D**) Body weight change analysis in Balb/c mouse THP-1 shRNA1896 xenograft models. Final body weight was compared in tumor-bearing animals receiving water contained Vehicle (10 ml/kg) and Dox (2mg/ml) with 5% sucrose. (**E**) Statistical analysis of anti-tumor growth effect in Balb/c mouse THP-1 shRNA1896 xenograft model.
